# Parent empowerment and coaching in early intervention: study protocol for a feasibility study

**DOI:** 10.1186/s40814-020-00568-3

**Published:** 2020-02-13

**Authors:** Melanie Pellecchia, Rinad S. Beidas, David S. Mandell, Carolyn C. Cannuscio, Carl J. Dunst, Aubyn C. Stahmer

**Affiliations:** 1 Penn Center for Mental Health, Department of Psychiatry, University of Pennsylvania Perelman School of Medicine, 3535 Market St. 3rd Floor, Philadelphia, PA 19104, 215-746-1950 USA; 2 Medical Ethics and Health Policy, University of Pennsylvania Perelman School of Medicine, Philadelphia, USA; 3 Penn Implementation Science Center at the Leonard Davis Institute (PISCE@LDI), University of Pennsylvania, Philadelphia, USA; 4 The Wharton School, University of Pennsylvania, Philadelphia, USA; 5 Orelena Hawk Puckett Institute, Asheville, USA; 6 Mind Institute, University of California Davis, Davis, USA

**Keywords:** Parent coaching, Autism spectrum disorder, Early intervention, Implementation toolkit

## Abstract

**Background:**

Parent-mediated early interventions (EI) for children with autism spectrum disorder (ASD) can result in significant improvements in children’s cognitive ability, social functioning, behavior, and adaptive skills, as well as improvements in parental self-efficacy and treatment engagement. The common component to efficacious parent-mediated early interventions for ASD is clinician use of parent coaching and occurs when a clinician actively teaches the parent techniques to improve their child’s functioning. Available evidence suggests that community-based EI clinicians rarely coach parents when working with families of these children, although specific barriers to coaching are unknown. This consistent finding points to the need to develop strategies to improve the use of parent coaching in community EI programs. The purpose of this community-partnered study is to iteratively develop and pilot test a toolkit of implementation strategies designed to increase EI clinicians’ use of parent coaching.

**Methods:**

This study has four related phases. Phase 1: examine how EI clinicians trained in Project ImPACT, an evidence-based parent-mediated intervention, coach parents of children with ASD. Phase 2: identify barriers and facilitators to clinician implementation of parent coaching by administering validated questionnaires to, and conducting semi-structured interviews with, clinicians, parents, and agency leaders. Phase 3: partner with a community advisory board to iteratively develop a toolkit of implementation strategies that addresses identified barriers and capitalizes on facilitators to improve clinician implementation of evidence-based parent coaching. Phase 4: pilot test the feasibility and effectiveness of the implementation strategy toolkit in improving EI clinicians’ use of parent coaching with nine EI clinicians and parent-child dyads using a multiple-baseline-across-participants single-case design.

**Discussion:**

Completion of these activities will lead to an in-depth understanding of EI clinicians’ implementation of parent coaching in usual practice following training in an evidence-based parent-mediated intervention, barriers to their implementation of parent coaching, a toolkit of implementation strategies developed through an iterative community-partnered process, and preliminary evidence regarding the potential for this toolkit to improve EI clinicians’ implementation of parent coaching. These pilot data will offer important direction for a larger evaluation of strategies to improve the use of parent coaching for young children with ASD.

## Background

Parent-mediated early intervention for children with ASD results in improved child outcomes across a range of developmental domains, as well as improved parental self-efficacy and treatment engagement [1–5]. We use the term “parent” throughout to refer to any primary caregiver of a child, including biological parents, guardians, and other familial and non-familial caregivers. The common component to efficacious parent-mediated early interventions for ASD is clinicians coaching parents. Parent coaching includes providing the parent the needed supports to improve their child’s skills and abilities through a structured system of jointly planning learning goals, modeling effective practices, and engaging in feedback [6]. Clinician use of evidence-based parent coaching is hypothesized to result in parent behavior change, which in turn leads to improved child outcomes.

Parent coaching in early childhood is an interactive process between a clinician and a parent that involves observation, reflection, and action to directly promote the parent’s ability to support his or her child’s participation in family and community activities [6–8]. Most evidence-based coaching models are based largely on adult learning theory, which posits that adults benefit from specific strategies to motivate and teach them [9]. Examples of parent coaching strategies based on the adult learning theory are provided in Table 1. Increasingly, leaders in early childhood education recognize that clinicians should shift from the traditional role of providing therapy directly to the child to enhancing parents’ efforts at improving the child’s participation in daily routines [8]. EI programs that encompass the entire family, not only the child, align with the family-centered practices recommended by the Division for Early Childhood of the Council for Exceptional Children for use in early intervention [10]. Consequently, there have been increased efforts to train clinicians in how to coach parents [11, 12].

**Table 1:** Examples of parent coaching strategies

Strategy	Description
Authentic learning experiences	Learning opportunities occur as part of real-life problems or challenges
Collaborative goal setting	Parent is actively involved in selecting goals and strategies for learning
Demonstration	Instructor models use of the technique through role-plays or actual application
In vivo feedback/guidance	Instructor observes parent’s use of strategies and provides immediate feedback
Reflection	Instructor engages parent in self-evaluation or assessment of performance

**Fig. 1:**
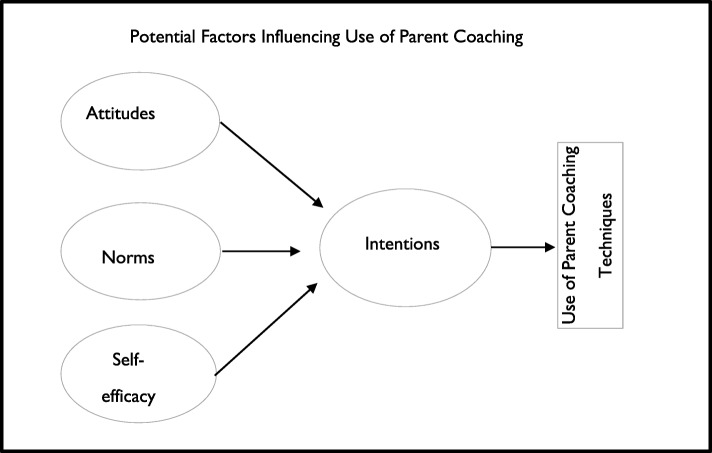
Conceptual model of EI clinician implementation using the theory of planned behavior

Despite these efforts, EI clinicians spend most of their time in traditional child-directed intervention, rather than in coaching parents [13, 14]. The reasons for poor implementation of parent coaching are unclear. Well-tested theories of behavior change can both provide insight into reasons EI clinicians do not use parent coaching and inform strategies to improve implementation. The theory of planned behavior posits that an individual’s intention to perform a certain behavior (in our case, use parent coaching) is the most proximal determinant of that behavior, when individuals have the ability to act on their intentions. Intentions are in turn influenced by three determinants: attitudes (e.g., whether one “likes” or “dislikes” using a given practice), norms (e.g., whether one perceives that using a given practice is expected by important others or whether one perceives that other similar practitioners use the practice), and self-efficacy (e.g., whether one believes that one has the necessary skills to perform the practice). This model is commonly used to predict health behaviors and has been used as a framework for understanding educators’ use of evidence-based practices (see Fig. 1) [15–17]. Previous work finds substantial variability in teachers’ and clinicians’ intentions to implement a new practice, and that intentions to implement a new practice are associated with its later implementation [16, 18]. Understanding EI clinicians’ intentions to implement parent coaching and whether these intentions are driven by attitudes, norms, and self-efficacy can lead to tailored implementation strategies that target specific mechanisms to increase use of parent coaching.

Regardless of the theory underlying implementation strategies, those developed in partnership with community stakeholders are more likely to be used than those that were developed without stakeholder input [19–21]. Successful implementation of evidence-based practices is most likely to occur when the implementation process is developed in partnership with the individuals who will use them [21–23]. Including community stakeholder perspectives in developing implementation strategies improves their ecological validity and ensures that they are consistent with the community’s priorities, culture, and values [24, 25]. Using community-academic partnerships (CAP) offers a novel approach to developing strategies to support the implementation and sustainment of parent coaching [26].

A new initiative in Philadelphia’s EI system presents a rare opportunity to observe and improve EI clinicians’ implementation of parent coaching with families of young children with ASD. Through this initiative, clinicians working within the EI service system will be trained in Project ImPACT [27], an evidence-based parent-mediated treatment for young children with autism. Pilot data suggest that Project ImPACT can be successfully implemented in publicly funded EI service systems [4, 28] and shows promise for wide-scale implementation. However, in each of these evaluations, Project ImPACT was implemented by EI clinicians who were actively seeking training and were highly motivated to learn the skills needed to use parent-mediated interventions. It is not clear how EI clinicians working in a service system that is planning a wide-scale implementation will incorporate the program into their existing practice. Previous studies have found significant variation in educators’ motivation to adopt new evidence-based practices within large-scale rollouts such as this one [18]. Associated barriers to implementing evidence-based practices are exacerbated in large under-resourced service settings, like the early intervention system in Philadelphia [29, 30]. This training initiative is an important step in training EI clinicians to coach parents of children with, or at risk for, ASD. However, large-scale implementation and sustainment of new initiatives often require efforts beyond training and coaching, including the use of thoughtfully executed implementation strategies. Implementation strategies are defined as “methods or techniques used to enhance the adoption, implementation, and sustainability of a clinical program or practice” [31] and encompass a wide range of strategies targeted toward systems, organizations, and individual clinicians [32]. The proposed study takes advantage of this rollout to conduct a systematic evaluation of potential barriers and facilitators to implementation, with the goal of developing strategies to support the successful implementation of parent coaching among clinicians trained in Project ImPACT.

## Methods

### Setting

In Philadelphia, children 36 months of age and younger with or at high risk for ASD are eligible for publicly funded intervention through the infant and toddler early intervention program. Thirteen agencies provide these assessment and early intervention services in Philadelphia. They use an interdisciplinary treatment approach, have a treatment philosophy that supports parents to engage therapeutically with their children, and provide home-based services. In 2016, these agencies served 814 children with autism. EI administration selected three of these agencies to participate in the initial training for Project ImPACT. The current study will assess use of Project ImPACT in those initial training sites. These agencies are representative of the broader service system with regard to the number of clinicians employed (mean = 43 per agency) and the number of children with/at risk for ASD they serve (mean = 35 per agency in 2018). All agencies in Philadelphia EI serve the entire Philadelphia County; therefore, there is little variability across agencies in terms of the families they serve. All providers receive standard training delivered by the county. All agencies employ similar staff from a limited pool of educational backgrounds, including educators, speech pathologists, and occupational therapists.

### Project ImPACT

ImPACT stands for Improving Parents as Communication Teachers. Project ImPACT is a naturalistic behavioral developmental intervention (NBDI) that includes (1) a curriculum to guide parents in supporting their child’s social and communication skills using naturally occurring interactions and (2) guidelines to help EI clinicians coach parents in the use of the intervention strategies. Project ImPACT uses methods that emphasize fostering the child’s relationship with others to develop reciprocity, social engagement, and shared affect during adult-child interactions while incorporating behavioral strategies (e.g., direct prompting, contingency reinforcement) during a highly engaged interaction to teach specific social communication skills [27]. The program is delivered individually to parents of young children with or at risk for ASD in their homes during weekly EI sessions. EI clinicians help parents select appropriate goals for their child and train them to use intervention techniques to achieve those goals. For example, a clinician might explain to the parent that a strategy for increasing communicative requests would be to obstruct access to preferred items by placing them out of reach, then model the technique and coach the parent in how to use it during daily routines. Prior to implementing Project ImPACT, clinicians receive two days of didactic training regarding use of the program’s intervention and coaching techniques. Training includes role-play and practice, followed by monthly case consultation calls for 6 months.

### Phase 1: Examine EI clinicians’ current use of parent coaching when working with parents of young children with ASD

The primary research question driving phase 1 is how do EI clinicians trained in Project ImPACT implement the parent coaching aspects of the intervention? We hypothesize that (1) there will be substantial variability among clinicians in their use of parent coaching, and (2) clinicians will use certain coaching techniques (e.g., modeling of strategies) more often than others (e.g., in vivo feedback).

#### Participants

We will recruit ten early intervention clinician/family dyads. EI clinicians will be recruited from the three publicly funded agencies participating in the Project ImPACT training initiative. Clinicians in these agencies are representative of clinicians in the broader service system with regard to professional background and children served on their caseloads. Recruitment will occur by randomly sampling without replacement from each agency, with the goal of recruiting evenly across the three agencies (i.e., 3–4 clinicians per agency). Inclusion criteria for EI clinicians will be as follows: (1) completed training in Project ImPACT prior to recruitment and (2) have at least three children with or at risk for ASD on his or her caseload. Families will be randomly selected from each EI clinician’s caseload. One family per clinician will be enrolled. Inclusion criteria for families will be as follows: (1) child that is less than 36 months of age; (2) child that has a classification of autism or high autism risk as determined by the EI system; (3) family that receives EI services through Philadelphia’s infant and toddler program; and (4) parent that speaks English or Spanish.

#### Setting

Observations will occur in the family’s home, the usual setting for the provision of family-based early intervention services. One intervention session (approximately 1 h) with each of the ten EI clinicians will be video recorded by the research team.

#### Measures

##### Clinician use of parent coaching

We will use the Project ImPACT Fidelity of Implementation for Coaching Form to measure EI clinicians’ fidelity to the parent coaching procedures outlined in the Project ImPACT manual [27]. This form is a 20-item observational tool that measures the clinician’s use of parent coaching techniques and has been used in a number of studies evaluating the effectiveness of Project ImPACT [4, 28, 33]. We will use the Triadic Intervention and Evaluation Rating Scale (TIERS) to measure EI clinicians’ use of collaborative coaching techniques. The TIERS is a validated observational tool designed to measure the use of parent-focused and collaborative coaching techniques within EI settings [34]. Both measures will be coded from video by the PI and trained coders.

#### Data analysis

Descriptive statistics will be used to depict and compare clinician behavior. Clinicians’ coaching fidelity will be calculated using the metrics described in each measure including the mean, range, and distribution of overall fidelity scores and clinicians’ fidelity to the individual components of the coaching fidelity measures. This will increase our understanding of whether clinicians are more likely to implement certain aspects of parent coaching than others. To ensure validity of the fidelity ratings, 20% of sessions will be coded by a second observer. Consistent with recommendations for direct collection of data within clinical research [35], point-by-point inter-observer agreement data will be calculated as percent agreement by dividing the number of agreements between observers by the sum of both agreements and disagreements and then multiplying by 100 to yield a percentage [36]. We will also calculate Cohen’s kappa to ensure our reliability metric meets the highest standards of accuracy.

### Phase 2: Identify barriers and facilitators using parent coaching in EI service systems

We will use a mixed methods approach to answer two related research questions in phase 2. First, we will use qualitative research methods to learn about the barriers and facilitators to EI clinicians’ implementation of parent coaching within a publically funded EI service system. We hypothesize that barriers and facilitators to implementation across several levels of implementation will be identified including contextual, organizational, and individual clinician factors. Second, we will use survey methods to learn about EI clinicians’ attitudes toward parent coaching, self-efficacy with implementing parent coaching, and intentions to implement parent coaching. Based on previous findings from the theory of planned behavior described above, we hypothesize that EI clinicians’ intentions to implement the components of parent coaching, as well as their attitudes, perceived norms, and ratings of self-efficacy, will vary across clinicians and across parent coaching techniques.

#### Participants

We will interview three agency leaders from each of the three agencies to learn about the extent to which parent coaching is expected, supported, and rewarded by agency leaders in EI. Inclusion criteria for agency leaders will be that they hold a leadership or supervisory role in an agency that employs EI clinicians trained in Project ImPACT. At least ten EI clinicians and ten parents (or the number needed to obtain saturation in the interviews), with the same inclusion criteria as described in phase 1, will be interviewed. The clinicians and parents interviewed for phase 2 may be the same sample observed in phase 1, if they are willing to participate in both observations and interviews.

#### Setting

Interviews will occur in the preferred location of each participant*.* This will likely include the clinician and agency leaders’ work site and the family’s home.

#### Measures

##### Qualitative interview data collection

We will use the theory of planned behavior to develop a semi-structured interview guide to learn about barriers and facilitators to parent coaching and the support needed to implement parent coaching in daily practice. We will query participants about (1) strategies EI clinicians report using during interactions with parents; (2) agency leaders’, clinicians’, and parents’ views about the acceptability and appropriateness of parent coaching within EI; and (3) contextual factors that may influence the procedures EI clinicians use (e.g., parent is too busy to participate). These interviews will provide textual data that can be analyzed for themes and patterns. Standardized probes will be included in the interview guide so that consistency across interviews is maintained. All interviews will be recorded and transcribed.

#### Qualitative data analysis

All interviews will be transcribed and imported into NVivo. Transcripts will be analyzed in an iterative process based upon an integrated approach that combines both priori questions and concepts derived inductively through close reading of the transcripts [37]. Members of the research team will develop a qualitative codebook through a collaborative and iterative process. First, the team will read through several interviews and search for major themes. Next, commonalities among observations will be discussed, and overlapping insights will be used to guide the initial framework for the codebook. The codebook will include operational definitions for each code and sample quotes. Coders will independently summarize key findings for each of the selected codes, including quotes that corroborate or diverge from the key findings. Finally, codes will be summarized and examined for patterns to develop theories about the data.

#### Measures of intention and determinants of intention

A questionnaire will include validated, standardized item stems to measure clinicians’ intentions, attitudes, norms, and self-efficacy regarding use of parent coaching. The stems for each question were designed to be adapted for study of any practice and have been used to successfully predict a large variety of practices [38, 39]. EI clinicians’ intentions to use parent coaching will be measured by items designed to specifically probe their intent to use the strategy (e.g., “How likely is it that you will coach parents of young children with/at-risk for ASD?”). Scaled response options will range from 1 (very unlikely) to 7 (very likely). Clinicians’ attitudes, or the extent to which one “likes” or “dislikes” using parent coaching strategies, will be measured by six items on a 7-point likert-type scale. For example, scales will allow respondents to rate using a parent coaching strategy as extremely useful to extremely un-useful and as extremely wise to extremely foolish. Clinicians’ perceived norms will be measured using standard questions that capture perceptions of normative pressure. For example, clinicians will be asked to rate on a 7-point scale the perception that most EI clinicians will use parent coaching strategies. Clinicians’ self-efficacy will be measured by asking respondents to rate, on a 7-point scale, the statement, “If I really wanted to, I could coach parents in my practice” as likely/unlikely. The survey will be administered to EI clinicians at the conclusion of the qualitative interview.

#### Mixed methods data analysis

We will integrate the qualitative findings with quantitative measures of clinicians’ intentions, and determinants of intention, and findings from the observations during phase 1. We will use mixed methods in two ways. First, we will use quantitative findings to identify patterns in the qualitative data by entering quantitative findings (e.g., clinicians’ attitudes) into Nvivo as attributes of each participant. Then, as themes emerge from the interviews, we will use Nvivo to query whether the presence and quality of these themes differ among clinicians. Second, we will use the qualitative data to help interpret quantitative results, especially if there are counterintuitive findings. For example, if intentions are generally high but the fidelity is low, we will use qualitative interviews to specifically query participants about why they believe that to be the case. These data will help identify the areas of greatest need for the implementation strategy toolkit based on the theory of planned behavior.

### Phase 3: Partner with community stakeholders to develop a toolkit of implementation strategies to improve EI clinicians’ implementation of parent coaching

We will use an iterative, community-partnered process to develop a toolkit of implementation strategies designed to improve the implementation of EI clinicians’ parent coaching. The toolkit will use the Patient Centered Oriented Research Institute (PCORI) dissemination and implementation toolkit [40] framework to develop the toolkit. This framework has been used to develop implementation toolkits to support the implementation of research-informed practices in community settings. Consistent with the PCORI framework, the toolkit will be informed by several community-partnered activities, including findings from the field observations, interview and survey data from phases 1 and 2, and discussions with a community advisory board (CAB), to ensure the toolkit is feasible for use in EI settings.

#### Participants

We will recruit an advisory board of 12 community stakeholders (4 EI administrators, 4 EI clinicians, and 4 parents of children with ASD) to guide the development of the implementation strategy toolkit. CAB members will be stakeholders from under-resourced communities served in Philadelphia. EI clinicians observed and interviewed during the activities in phases 1 and 2 will be excluded. Parents may be currently or recently served by the Pennsylvania Part C system (a federally funded program that provides early intervention services for children under 3 years of age). All other inclusion criteria will be the same as described in phases 1 and 2.

#### Community advisory board meetings

The toolkit will be developed through an iterative process of CAB meetings involving a mutual sharing of expertise and shared decision-making [41]. Consistent with other uses of community academic partnerships to inform intervention development [42, 43] meetings will include knowledge sharing, such as educating stakeholders regarding evidence-based practices for ASD (including the findings from aims 1 and 2), and stakeholders educating the research team regarding the community’s resources, needs, and priorities. The CAB will meet eight times, beginning while the field observations are being conducted during phase 1 and throughout the pilot study in phase 4. A description of topics planned for the CAB meetings is provided in Table 2. CAB meetings will include information about the community’s priorities and needs around parent coaching, the Part C service system infrastructure for supporting parent coaching, and the support for specific implementation strategies. The CAB will provide input into the development of the implementation toolkit from initial formulation through the final draft to ensure it is feasible and acceptable for use in the Part C system, including all aspects of the manual development such as content and formatting, to ensure that it is user-friendly and easily adopted by community clinicians. We will then meet with the CAB after the pilot study to make any needed revisions based on the pilot study findings.

**Table 2:** Topics planned for CAB meetings

CAB meeting 1	Introductory meeting. Review project purpose and partnership plans. Discuss current service procedures and priorities for clinicians and families of young children with ASD within the EI system.
CAB meeting 2	Present findings from the field observations, interviews, and surveys. Gather input about how these findings may or may not be reflective of EI system’s usual practices.
CAB meeting 3	Present theory of planned behavior and potential strategies that may fit the needs of the service setting. Obtain CAB feedback about the potential strategies that may be included in the toolkit.
CAB meeting 4	Begin to outline the toolkit. Discuss implementation strategies that may be feasible and acceptable by stakeholders.
CAB meeting 5	Develop toolkit content including finalizing the implementation strategies to be included in the toolkit and detailed descriptions of procedures for using those strategies.
CAB meeting 6	Discuss the toolkit content, including formats to display the content (checklists, handouts, vignettes, etc.). CAB offers guidance on formatting tools so they are feasible and easily accessible.
CAB meeting 7	Discuss refinements to the toolkit so that it is ready for use in the pilot study.
CAB meeting 8	Discuss findings from the pilot study and potential adaptations to the toolkit based on findings. Discuss plans for continued collaboration and implementation by EI clinicians.

#### Content of the toolkit

**Fig. 2:**
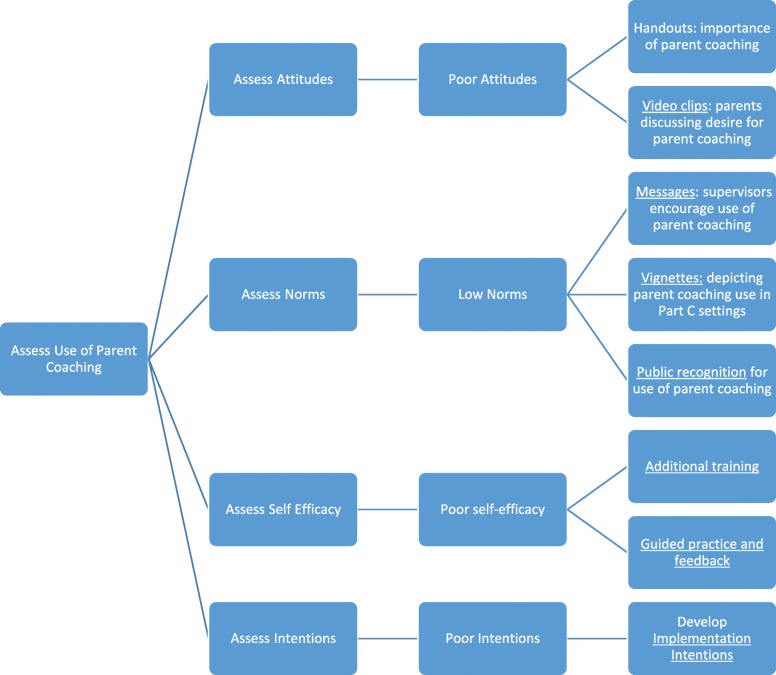
Potential process for selecting implementation strategies in the providers successfully partnering with parents toolkit

The toolkit, potentially named *Providers Successfully Partnering with Parents*, will include implementation strategies to address the barriers identified during Aims 1 and 2; therefore, the exact content of the toolkit is not known. We expect the toolkit will likely include strategies to improve EI clinicians’ self-efficacy for parent coaching, attitudes toward parent coaching, feelings of normative pressure to implement parent coaching, and address contextual barriers. Organizational and system-level barriers to implementation may arise, but are beyond the scope of this pilot study and will inform further adaptations of the toolkit. Examples of implementation strategies that could potentially be included in the toolkit are listed in Table 3. Clear and concrete definitions of each implementation strategy will be included in the toolkit. Each strategy will have its own chapter detailing concrete examples, plans for responding to potential barriers to its use, vignettes depicting its use in EI settings, handouts, and visual supports to aide use of implementation strategy. The toolkit will include many examples, checklists, handouts, and graphics to ensure that individuals with varying experience and skill can use it. The CAB will be the key for developing examples and integration of strategies to make them relevant to EI providers. A graphic displaying the potential process of selecting implementation strategies in the toolkit is provided in Fig. 2.

**Table 3:** Sample implementation strategies for potential inclusion in the clinicians successfully partnering with parents toolkit

Implementation barrier	Potential strategies
Poor clinician self-efficacy (i.e., belief that one does not have the skills or ability to perform some aspect of parent coaching)	o Additional training in parent coaching o Guided practice and feedback to improve skills and self-perception of competency
Poor clinician attitudes (i.e., negative beliefs about the outcomes of performing some aspect of parent coaching)	o Handouts describing rationale and importance of parent coaching in EI o Video clips of parents discussing their desire for parent coaching
Low normative pressure (i.e., the belief that supervisors or other important persons don’t expect implementation of aspects of parent coaching, or that other clinicians like them will not implement parent coaching)	o Regular messages sent via text communicating that supervisors encourage the use of parent coaching and/or that other EI clinicians are using parent coaching o Vignettes depicting parent coaching use in Part C settings o Public recognition of EI clinicians who implement parent coaching with high fidelity
Environmental constraints (i.e., barriers that interfere with the implementation of parent coaching despite strong intentions to perform some aspect of parent coaching)	o Communicating strategies to EI clinicians that can be used to decrease interruptions during parent coaching sessions (e.g., asking parents for a dedicated time, asking parents to turn off their cell phones, finding a quieter room in the house, setting siblings up with toys of a video before beginning parent coaching)

### Phase 4: Pilot test the feasibility, promise, and acceptability of the implementation strategy toolkit with dyads of EI clinicians and parents of children with or at risk for ASD

We will use a single-case multiple baseline across participants deign to pilot test whether or not improvements in clinician coaching fidelity, parent use of ImPACT intervention strategies, and child outcomes are observed following introduction of the implementation toolkit.

#### Participants

We will recruit nine EI clinicians (3 from each participating agency) and nine parent-child dyads (one dyad randomly selected from each clinician’s caseload) to participate in the pilot test. Clinicians will have completed training in Project ImPACT at least one month prior to recruitment to ensure some experience with the program. Parents with children under 30 months of age will be recruited to ensure that the child does not age out of the EI service system (at 36 months) before completing the study. All other inclusion criteria for clinicians and parents will be identical to phase 1.

#### Setting

The pilot test study will occur during the family’s usual EI sessions, likely in the family’s home.

#### Measures

##### EI clinicians’ parent coaching fidelity

EI clinicians’ parent coaching fidelity will be rated using the Project ImPACT Fidelity of Implementation for Coaching Form [27] each session. A trained research assistant blind to the timing of the recording (i.e., baseline or intervention phase) will code parent coaching fidelity data from video-recorded observations of the usual sessions.

##### Parents’ strategy use

Parents’ use of the treatment strategies will be measured using procedures consistent with previous evaluations of Project ImPACT [33]. At the start of each EI session, parents will be asked to interact with their child for 10 min in the way they typically would during play. Parent behavior will be video recorded and scored (by trained raters blind to the study condition) for correct use of the intervention strategies using the Project ImPACT Fidelity of Intervention Implementation Form [27]. Each of six parental strategies will be scored on a scale from 1 (“Parent does not implement throughout session”) to 5 (“Parent implements throughout session”) and then averaged to compute an overall fidelity rating for each routine. Ratings for each item will be averaged to compute an overall fidelity rating.

##### Children’s social communication

Changes in children’s social communication skills will be measured using the Brief Observation of Social Communication Change, [44] scoring applied to the parent-child interaction videos. The BOSCC is a recently developed observational coding scheme designed to be sensitive to changes in social communication behavior, easily used by naïve, minimally trained examiners, and coded relatively quickly. The BOSCC has strong interrater and test-retest reliability, sensitivity to change, and evidence of convergent and discriminative validity [45]. For all video recorded and coded data, at least 20% will be coded by a second observer balanced across experimental conditions (i.e., baseline and intervention phases) and participants [46]. Point-by-point inter-observer agreement data will be calculated to evaluate the validity of all data [37].

##### Treatment acceptability

Clinicians’ acceptability of the toolkit will be assessed at the end of intervention using a brief 12-item scale designed to assess acceptability of new practices using a 5-point Likert scale.

##### Feasibility

Feasibility of the study procedures will be assessed based on whether we meet recruitment milestones and the percentage of families enrolled from those recruited. We will also assess feasibility through family and provider attrition from the study in order to gather information on the percentage of families and providers who complete the intervention.

### Design and analysis

A multiple-baseline design across participants will be used to evaluate changes in EI clinicians’ parent coaching fidelity as a function of the introduction of the implementation strategy toolkit. Single-case research design is ideal for studies with small samples and allows for rigorous analysis of intervention effects [46]. The multiple baseline design to be used in the pilot study is an established method to systematically evaluate intervention effects and allows for causal inferences [44]. The baseline phases of the design will consist of Project ImPACT without the implementation toolkit. The intervention phases in the design will be consistent of Project ImPACT + the implementation toolkit. We will evaluate change in provider coaching fidelity within the pilot study as the primary outcome of interest following the introduction of the toolkit, using the multiple baseline design procedures. We will also collect secondary outcome data regarding changes in parent’s use of the intervention techniques and children’s change in social communication. These secondary outcome data are exploratory and will allow us to gather some preliminary data regarding any pre- and post-changes to child and family outcomes observed following changes in provider coaching.

The multiple baseline design is ideal because it enables more valid causal inferences by staggering the intervention sequentially across one clinician at a time [46]. Consistent with the single-case intervention research design standards [46], the introduction of and training in the implementation strategy toolkit will be staggered across EI clinicians within agencies. The implementation strategy toolkit will be introduced with each subsequent clinician when the preceding clinician attains at least 80% coaching fidelity or fidelity stabilizes across three sessions, until the toolkit has been introduced across all three clinicians within each agency. The same procedure will be used for all three agencies. Three clinicians per agency will allow for an adequate demonstration of phase repetitions to infer causal relationships between the independent and dependent variables [46]. Progression through the phases of the multiple baseline design is dependent upon the change in EI clinicians’ parent coaching fidelity. We will visually analyze the data independently along two dimensions: (1) within phases—to evaluate level, trend, and variability of the data points and (2) across phases—to evaluate immediacy of effect, overlap, and consistency of data in similar phases. This will allow us to assess whether there are at least three demonstrations of effect across three different points in time and make causal inferences about any observed changes in EI clinician parent coaching fidelity [46]. The primary outcome of interest is EI clinician fidelity to the Project ImPACT Fidelity of Implementation for Coaching Form, as this outcome is most proximally related to the implementation strategy toolkit. Progression through the multiple baseline design phases will be contingent upon changes in provider coaching fidelity. In the event that a provider does not attain 80% coaching fidelity after the toolkit is introduced, a contingency plan consistent with best practices in single-case intervention research design standards will be adopted [47]. Each provider’s coaching fidelity data will be graphed and visually analyzed following each session to evaluate level, trend, and variability of the data points. If these analyses indicate that a provider’s coaching fidelity is (1) stable and (2) not demonstrating an increasing trend over three consecutive sessions, the implementation toolkit will be introduced with the subsequent provider in the multiple baseline design, while continuing to intervene and monitor coaching fidelity with the previous provider. Secondary outcomes include changes in parent’s use of the treatment strategies and children’s social communication. These secondary outcomes will be recorded and analyzed prior to and following the intervention to evaluate changes in either of these distal outcomes as a function of the introduction of the implementation strategy toolkit.

## Discussion

Several models of parent-mediated interventions for young children with ASD have been shown to improve child and parent outcomes [1–5]. Each of these models includes the use of parent coaching to actively transfer skills from a clinician to the parent. However, preliminary evidence suggests that clinicians working in community settings rarely adopt parent coaching techniques with families of young children with ASD. This study will use a community-partnered iterative process to develop and pilot test a toolkit of implementation strategies to improve EI clinicians’ use of parent coaching for families of young children with ASD in community settings. Completion of the activities in this pilot study will lead to (1) an in-depth understanding of EI clinicians’ implementation of parent coaching in usual practice following training in an evidence-based parent-mediated intervention; (2) barriers to their implementation of parent coaching; (3) a toolkit of implementation strategies developed through an iterative community-partnered process; and (4) preliminary evidence regarding the potential for this toolkit to improve EI clinicians’ implementation of parent coaching. These research findings will lay the foundation for a later proposal to test the effectiveness of the toolkit in a randomized trial.

The implementation strategies developed through this study will be contextually relevant to the Philadelphia EI setting and tailored to each individual clinicians’ needs. For example, implementation strategies for a clinician with poor use of parent coaching due to poor attitudes toward coaching (i.e., prefers a more child-directed approach to treatment) may include infographics describing the benefits of parent coaching or exposure to vignettes of other clinicians reporting favorable views toward parent coaching. Conversely, implementation strategies for a clinician with poor use of parent coaching due to poor self-efficacy may include additional guided practice and feedback. This individualized approach to selecting implementation strategies for each clinician is likely to improve the effectiveness of the implementation toolkit and offers important insights into the feasibility of individualizing implementation strategies for clinicians within a larger system. Future research should expand upon this line of work and include an in-depth examination of organizational and system-level barriers to implementing parent coaching within early intervention and developing implementation strategies to address those barriers.

## Conclusions

The study described in this paper encompasses several important innovations for the implementation of evidence-based treatment models for young children with ASD in community settings. First, this study is the first to study the use of parent coaching strategies for young children with ASD in community settings, rather than autism interventions per se, which is important because parent coaching is likely the active mechanism of change within parent-mediated interventions. Tools developed to support the use of parent coaching can likely be applied to many intervention models and improve the implementation of community-based autism treatment more broadly. Second, despite growing evidence to support the use of parent-mediated interventions for young children with ASD, little attention has been given to the actual use, and barriers and facilitators to using these approaches, within community-based treatment setttings. This study will provide important insights into the challenges of implementing evidence-based treatments for young children with ASD in community settings and potential strategies to overcome these challenges. Finally, this research study relies heavily on a partnership with the EI system and uses iterative community-partnered research methods to inform the approach throughout all phases of the project. This community-partnered approach will improve the ecological validity of the strategies developed through this study and will ensure that they are consistent with the community’s needs and priorities [24, 25]. The study described in this paper provides valuable insights into strategies to support the implementation of evidence-based practices for young children with ASD in community settings.

## Data Availability

Not applicable

## Abbreviations

EI: Early intervention
ASD: Autism spectrum disorder
